# Regulation of the *kiss2* promoter in yellowtail clownfish (*Amphiprion clarkii*) by cortisol *via* GRE-dependent GR pathway

**DOI:** 10.3389/fendo.2022.902737

**Published:** 2022-08-03

**Authors:** Shao-Yang Bu, Yan-Yu Zhang, Xian Zhang, Tian-Xiu Li, De-Cai Zheng, Ze-Xiang Huang, Qian Wang

**Affiliations:** Department of Aquaculture, College of Marine Sciences, Hainan University, Haikou, China

**Keywords:** cortisol, stress, *kiss2* promoter, glucocorticoid receptor, *Amphiprion clarkii*

## Abstract

Kisspeptin plays a vital role in mediating the stress-induced reproductive regulation. Cortisol, known as a stress-related hormone, is involved in gonadal development and sexual differentiation by binding with glucocorticoid receptor (GR) to regulate the expression of *kiss* gene. In the present study, cortisol treatment in yellowtail clownfish (*Amphiprion clarkii*) showed that the expression of *kiss* (*kiss1* and *kiss2*) and *gr* (*gr1* and *gr2*) genes were increased significantly. We demonstrated that the yellowtail clownfish Kiss neurons co-express the glucocorticoid receptors in the telencephalon, mesencephalon, cerebellum, and hypothalamus. We further cloned the promoter of *kiss2* gene in yellowtail clownfish and identified the presence of putative binding sites for glucocorticoid receptors, estrogen receptors, androgen receptors, progesterone receptors, AP1, and C/EBP. Applying transient transfection in HEK293T cells of the yellowtail clownfish *kiss2* promoter, cortisol (dexamethasone) treatment was shown to enhance the promoter activities of the yellowtail clownfish *kiss2* gene in the presence of GRs. Deletion analysis of *kiss2* promoter indicated that cortisol-induced promoter activities were located between position −660 and −433 with GR1, and −912 and −775 with GR2, respectively. Finally, point mutation studies on the *kiss2* promoter showed that cortisol-stimulated promoter activity was mediated by one GRE site located at position −573 in the presence of GR1 and by each GRE site located at position −883, −860, −851, and −843 in the presence of GR2. Results of the present study provide novel evidence that cortisol could regulate the transcription of *kiss2* gene in the yellowtail clownfish *via* GRE-dependent GR pathway.

## Introduction

Kisspeptin is regarded as the key factor of reproduction and plays a role in the hypothalamus–pituitary–gonad axis (HPG axis) in vertebrates (1, 2). Kisspeptin binds to its receptor (G protein-coupled receptor 54, GPR54) and releases gonadotropin-releasing hormone (GnRH) from the hypothalamus, thereby stimulating the secretion of the gonadotropic hormone (GtH). Knockout of *Kiss1/Gpr54* in mice has been shown to prevent sexual maturity, cause gonad hypoplasia, hypogonadotropic hypogonadism, and infertility (3, 4). Moreover, kisspeptin has been reported to be involved in a variety of physiological activities, such as glucose homeostasis and light signal regulation (5, 6). Especially, kisspeptin is supposed to be involved regulation of various hormones on the hypothalamus–pituitary–adrenal axis (HPA axis) and the HPG axis under stress induction (7, 8).

Stress can lead to the dysfunction of the HPG axis and reproductive behavior through the HPA axis in vertebrates (9). There is a close relationship between stress and reproductive disorders (10). In patients with depression under stress, an excessive corticotropin-releasing hormone (CRH) level leads to the inhibition of the HPG axis, and increased cortisol level further inhibits the action of GnRH neurons, luteinizing hormone (LH) amplitude, follicle-stimulating hormone (FSH) levels, and LH pulse frequency (11–13). In mice, both psychosocial stress and unpredictable chronic stress reduce the expression of hypothalamic *kiss1* and the activity of kisspeptin neuron (14, 15). Moreover, corticotropin-releasing hormone or corticosterone treatment suppress *kiss1* expression and kisspeptin neuron activity in the brain of female rats and mice (16, 17). Glucocorticoid, a steroid regulated by stress, can bind with glucocorticoid receptor (GR) to regulate gene expression by associating with specific genomic glucocorticoid response elements (GREs) (18). In female rats, GR protein is detected in the kisspeptin neurons of periventricular nucleus continuum (AVPV/PeN) and arcuate nucleus (ARC), demonstrating that kisspeptin neurons can be modulated directly by glucocorticoid *via* GR (19). Additionally, GRE is found in the promoter regions of *kiss* genes in goldfish (*Carassius auratus*) (20). However, the molecular mechanism of glucocorticoid-regulated *kiss* genes in vertebrate species is still unknown.

Kisspeptin could be encoded by multiple genes in non-mammalian, and two paralogous *kiss* genes, known as *kiss1* and *kiss2*, are found in some teleosts, such as zebrafish and medaka (2). The yellowtail clownfish (*Amphiprion clarkii*) is a protandrous teleost whose sex is associated with social status within a group, including a male–female breeding pair and some non-breeders (21, 22). In one social unit, females occupy the first dominant status and inhibited sexual development of subdominant male and non-breeders, and subdominant male could undergo sexual development to female after female disappeared or the largest non-breeder change sex to male after the disappearance of male (22, 23). The level of cortisol, the main component of glucocorticoid in teleosts, depends on their social status in the population. In the protogynous orange-spotted grouper (*Epinephelus coioides*), the female treated with cortisol will change to male (24). In our previous research, the higher levels of hypothalamic *kiss2*/*gr2* expression and gonadal hormone were found in the subordinate of yellowtail clownfish (25, 26). Moreover, there is the sexually dimorphic distribution of *kiss1* and *kiss2* in the brain of yellowtail clownfish, especially in dorsal habenular nucleus (NHd) and dorsal part of the nucleus of the lateral recess (NRLd), which are involved in the regulation of reproductive function and environmental cues (27).

In order to better understand how cortisol exerts its action on the expression of *kiss* gene *via* the GR and thereafter regulates the reproduction in yellowtail clownfish, the expression of *kiss* and *gr* genes were examined after cortisol treatment. Moreover, the co-localization of *gr1*, *gr2*, and *kiss1*/*kiss2* mRNA were also studied in the brain by RNAscope. Then, the promoter region of *kiss2* was cloned by genome walking and predicted with the online tool for potential GR binding sites. In addition, after cortisol treatment, the kisspeptin promoter activities were detected in HEK-293T cells expressing yellowtail clownfish GR1 or GR2. Finally, the regulatory regions and binding sites of GR were identified by deletion analysis and site-directed mutagenesis analysis.

## Materials and methods

### Animals

Sexually mature and 3-month-old immature yellowtail clownfish were purchased from a local aquarium market (Dongfang city, Hainan, China) in June of 2021. The fish, nine per group, were reared in glass tank (length, 45 cm; width, 35 cm; and height, 60 cm) with continuously flowing aerated seawater at 27 ± 1°C. The photoperiod was a 12:12-h light–dark cycle, with lights turn on at 07:00 and off at 19:00. The fish were fed with commercial feed twice a day (09:00 and 18:00) and reared for a period of 1 week before experiment.

All animals used in this study were conducted in accordance with the guidelines of the animal welfare of the National Committee and approval of the Institutional Animal Care and Use Committee of Hainan University (HNUAUCC-2021-00014).

### Experimental design and sampling

The cortisol (hydrocortisone 21-hemisuccinate; MCE, NJ, USA) was dissolved in dimethyl sulfoxide (DMSO) and then diluted in the ratio of 10% cortisol, 40% polyethylene glycol 300 (MCE, NJ, USA), and 50% saline (0.9%). The immature fish (length, 4 ± 0.5 cm; weight, 2 ± 0.5 g) were anesthetized with MS-222 (Sigma, MO, USA) and then given an intraperitoneal injection with cortisol (10 or 50 mg/g body mass) at a volume of 10 μl/g body mass. The control group was injected the same liquid but without cortisol. We collected the whole brain at 6, 12, 24, and 48 h after injection, respectively.

The gonads of sexually mature yellowtail clownfish (length, 11 ± 1 cm; weight, 21 ± 5 g) were isolated and fixed in Bouin’s solution (Sigma, MO, USA) after anesthesia. The gonadal tissues were embedded in paraffin and cut into 5-μm paraffin sections for indentation of gonadal development. The brain of fish was fixed in 4% paraformaldehyde fix solution (Sigma, MO, USA) for *in situ* hybridization.

### RNA extraction, reverse transcription, and quantitative real-time PCR

Total RNA from the whole brain were extracted by the TRIzol method and then was reversed transcribed into cDNA using the HiScript II 1st Strand cDNA Synthesis Kit (Vazyme, Nanjing, China). Primer sequences and primer efficiency for quantitative PCR (qPCR) are listed in **Supplementary Table S1**. The quantitative real-time PCR was performed by Roche Light Cycler 96 real-time PCR System using ChamQ Universal SYBR qPCR Master Mix (Vazyme, Nanjing, China) according to the manufacturer’s protocol. The qPCR program was as follows: denaturation at 95°C for 30 s, then followed by 40 cycles at 95°C for 5 s and 55–58°C for 30 s and 72°C for 30 s. Each sample was used in triplicate. The relative mRNA levels of *kiss1*, *kiss2*, *gr1*, and *gr2* were evaluated using comparative threshold cycle (Ct) method with *β*-actin as internal reference gene and then calculated with the formula 2^−ΔΔCt^ (28).

### RNAscope *in situ* hybridization

Brain samples of female yellowtail clownfish were fixed in 4% paraformaldehyde at 4°C overnight. Samples successively were immersed in 10%, 20%, and 30% sucrose containing phosphate-buffered saline (PBS) for dehydration. Cross-sections (10 μm) were generated after being frozen on dry ice in optimal cutting temperature (OCT) compound. RNAscope *in situ* hybridization was performed following the manufacturer’s protocol from Advanced Cell Diagnostics (ACD). All steps demanding incubation at 40°C were achieved in the HybEZ Oven (ACD, Hayward USA). Binding of the specific probes against *kiss1* (1044931), *kiss2* (1044941), *gr1* (1088191), and *gr2* (1088201) were detected with RNAscope^@^ Multiplex Fluorescent Reagent Kit v2 (ACD, Hayward, USA). Probes *actb2* (1045881) and *dapB* (310043) were used as positive and negative controls, respectively. Images were taken by Nikon ECLIPSE Ti2 (Nikon, NY, USA).

### Cloning *kiss2* promotor

Genomic DNA was extracted from yellowtail clownfish muscle using phenol-chloroform methods. The 5′-flanking region of the *kiss2* was isolated in the reference to Universal Genome Walker Kit (Takara, Tokyo, Japan). The gene-specific primers were designed in the exon 1 based on the sequences of yellowtail clownfish *kiss2* (GenBank: MK368702.1) and are shown in **Supplementary Table S1**. Products of primary PCR were diluted 100 times, and then, secondary PCR was performed with the diluted products as the template. The secondary PCR products were purified using FastPure Plasmid Mini Kit (Vazyme, Nanjing, China) and were subcloned into the pMD 19-T Vector (Takara, Tokyo, Japan) for sequencing. The transcriptional start site (TSS) of *kiss2* was determined by our previous result of 5′-rapid amplification of cDNA ends. Transcription factor binding sites were predicted using the online PROMO (http://alggen.lsi.upc.es/recerca/menu_recerca.html) and gene-regulation tool (http://gene-regulation.com/cgi-bin/pub/programs/alibaba2/webbaba2.cgi).

### Construction of recombinant vector

A 1,442-bp 5′-flanking region and 48-bp exon 1 of *kiss2* was obtained from a pair of primers containing two different restriction enzyme sites, respectively, namely, KpnI and XhoI. PCR was performed with PrimeSTAR HS DNA Polymerase (Takara, Tokyo, Japan). The PCR products and pGL4.10 vector (Promega, WI, USA) were digested by KpnI and XhoI restriction endonucleases (NEB, MA, USA). After purification, the digested products were ligated using T4 DNA Ligase Kit (NEB, MA, USA). The construction of recombinant vector above, namely, pkiss2-1442, was used as template to construct a series of deletion vectors, namely, pkiss2-912, pkiss2-775, pkiss2-660, pkiss2-433, and pkiss2-335. All constructs were sequenced ensuring accuracy. The recombinant vectors were extracted with Omega Endo-Free plasmid DNA mini kit II (OMEGA, GA, USA). Primers used in here are presented in **Supplementary Table S1**.

### Cell culture, transient transfections, and luciferase assays

HEK-293T cells (Bosterbio, CA, USA) were cultured in Dulbecco’s modified Eagle’s medium (DMEM) containing 10% fetal bovine serum at 37°C with 5% CO_2_ and passed at least two generations prior to transfection. The healthy cells were seeded into 48-well plates, and approximately 1.5 × 10^5^ cells/well were cultured for 12 h. Cells were then co-transfected with 0.5 μg pkiss2-1442/pkiss2-912/pkiss2-775/pkiss2-660/pkiss2-433/pkiss2-335, 0.05 μg pcDNA3.1-GR1/pcDNA3.1-GR2 (yellowtail clownfish glucocorticoid receptor expression plasmid) and 0.025 μg pRL-CMV in 250 μl Opti-MEM using EZ Trans (Liji, Shanghai, China). After transfection of 6 h, cells were treated with 10^−7^ M dexamethasone sodium (DXMS, exogenous cortisol). Luciferase activities were detected 24 h later with Dual Luciferase Kit (Promega, WI, USA) in GloMax Discover (Promega, WI, USA).

### Site-directed mutagenesis

Mutations of putative GRE sites in the *kiss2* promoter were carried out using a series of specific primers (**Supplementary Table S1**) by two rounds of PCR amplification. Briefly, primers containing restriction enzyme site and mutation point were used to amplify the mutated fragments of upstream and downstream from the mutation site. The first-round PCR conditions were as follows: denaturation at 95°C for 30 s, followed by 40 cycles at 95°C for 5 s and 67–69°C for 30 s and 72°C for 1 min, with the final extension at 72°C for 10 min. The two PCR products were purified and mixed together for the second-round PCR. The conditions were as follows: denaturation at 95°C for 30 s, then followed by 9 cycles at 95°C for 5 s and 67–69°C for 30 s and 72°C for 1 min and 30 s. Then, primers for full-length promoter amplification of *kiss2*-1442-F and *kiss2*-1442-R were added and continued for an additional 20 cycles at 95°C for 5 s and 69°C for 30 s and 72°C for 1 min 30 s, with the final extension at 72°C for 10 min. PCR products were digested by KpnI and XhoI restriction endonucleases and were subcloned into pGL4.10 vector. All constructs were sequenced ensuring accuracy, and plasmid DNAs were extracted with Omega Endo-Free plasmid DNA mini kit II.

### Statistical analysis

All data are shown as mean ± standard error of the mean (SEM). Statistical analysis was performed using one-way ANOVA followed by Tukey’s multiple comparisons test in GraphPad Prism 7.0 (GraphPad Software, SD, USA). Results were considered significantly different when *p*-value was <0.05 (*p* < 0.05).

## Results

### Effects of cortisol on yellowtail clownfish *kiss1*, *kiss2*, *gr1*, and *gr2* expression profiles

**Figure 1:**
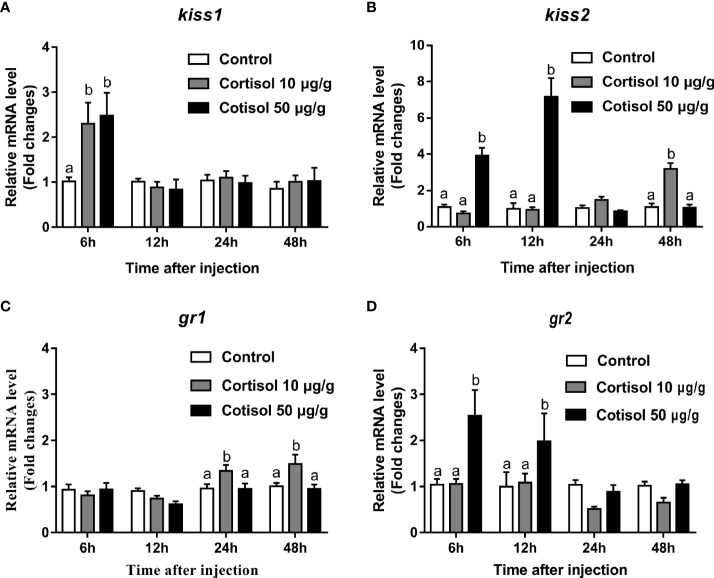
Effects of cortisol on *kiss1*, *kiss2*, *gr1*, and *gr2* mRNA levels in the brain of yellowtail clownfish. Expression of *kiss1* **(A)**, *kiss2* **(B)**, *gr1* **(C)**, and *gr2* **(D)** after injection of cortisol with both concentrations at 10 and 50 μg/g during 48 h. The data are expressed as mean ± SEM (n = 5–8). Bars with different letters indicate significant differences between treatments at the same sampling time (p < 0.05).

After cortisol injection, real-time PCR was performed to investigate the expression profiles of *kiss1*, *kiss2*, *gr1*, and *gr2* in the brain of immature yellowtail clownfish. The highest *kiss1* levels were detected at cortisol treatment with concentrations of 10 and 50 μg/g after 6 h (**Figure 1A**). For *kiss2*, transcripts were elevated 2-fold at 6 h and 3.5-fold at 12 h in the 50 μg/g cortisol-treated group relative to the other groups and were highest at 48 h in the 10 μg/g cortisol treatment (**Figure 1B**). The *gr1* mRNA levels were significantly higher at 24 and 48 h cortisol treatment with a dose of 10 μg/g (**Figure 1C**), but the most abundant *gr2* transcripts were at 6 and 12 h cortisol treatment with 50 μg/g (**Figure 1D**). These results indicated that cortisol treatment enhanced the transcription of *kiss1* and *kiss2* and *gr1* and *gr2* in the brain of yellowtail clownfish.

### Co-expression of *gr1* and *gr2* and *kiss1*/*kiss2* genes

**Figure 2:**
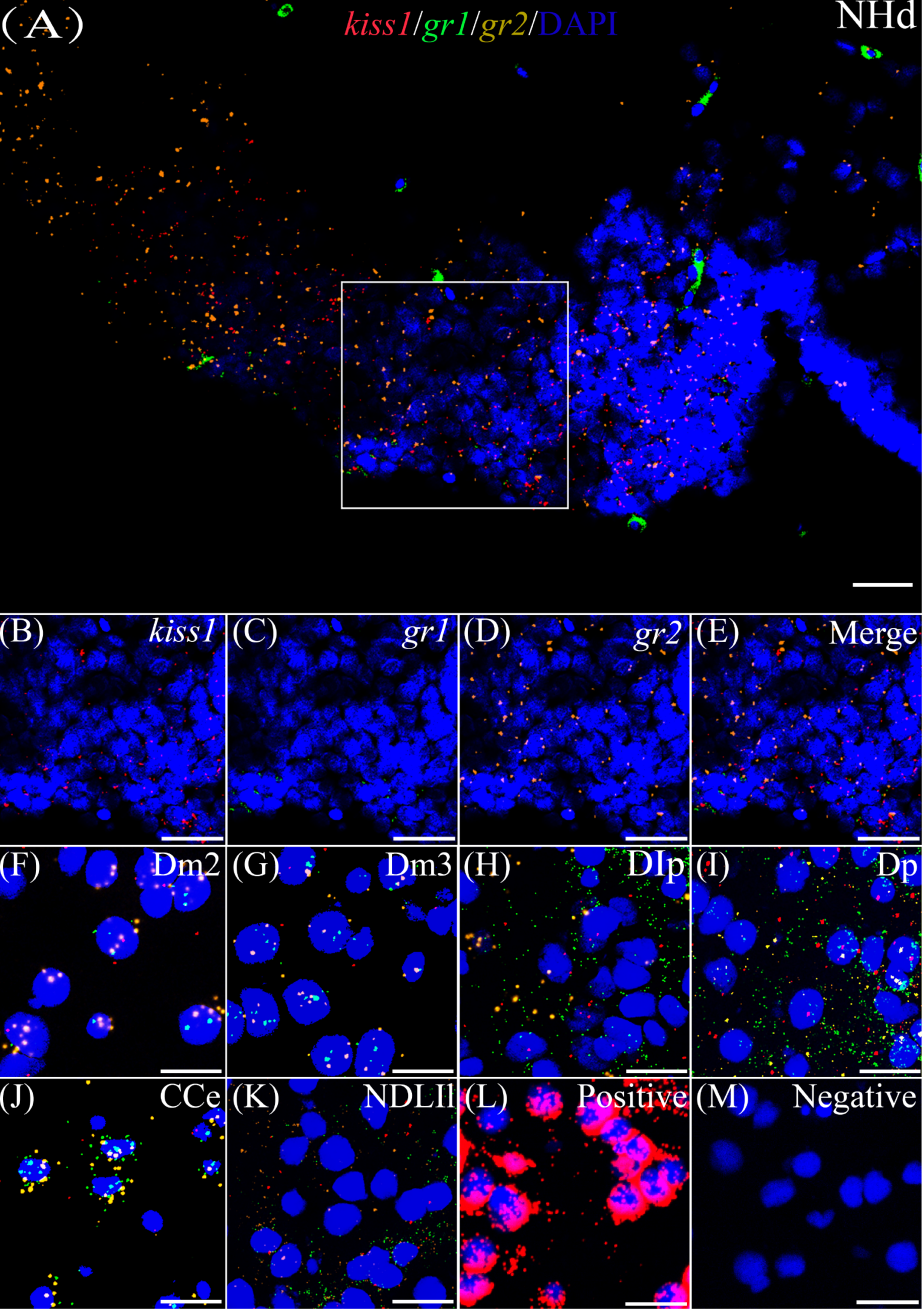
Co-expression of *gr1*, *gr2*, and *kiss1*. The view of NHd **(A)** shows *kiss1* (red), *gr1* (green), and *gr2* (yellow) with DAPI cell nuclear staining (blue). **(B–E)** The view of boxed region in panel **(A)**, showing *kiss1* (red), *gr1* (green), and *gr2* (yellow) with DAPI cell nuclear staining (blue) in panels **(B–D)**, respectively, and the “merge” in panel **(E)** shows *kiss1* (red), *gr1* (green), and *gr2* (yellow) with DAPI cell nuclear staining (blue). Representative images display *gr1* (green) and *gr2* (yellow) co-expression of *kiss1* (red) with DAPI cell nuclear staining (blue) in Dm2 **(F)**, Dm3 **(G)**, DIp **(H)**, Dp **(I)**, CCe **(J)**, and NDLIl **(K)**. Positive and negative controls are shown in panels **(L, M)**, respectively. NHd, dorsal habenular nucleus; Dm2, subdivision 2 of the medial dorsal telencephalic area; Dm3, subdivision 3 of the medial dorsal telencephalic area; DIp, lateral posterior part of the dorsal telencephalic area; Dp, posterior portion of the dorsal telencephalon; CCe, corpus of the cerebellum; NDLIl, lateral part of the diffuse nucleus of the inferior lobe. Bars = 20 μm.

**Figure 3:**
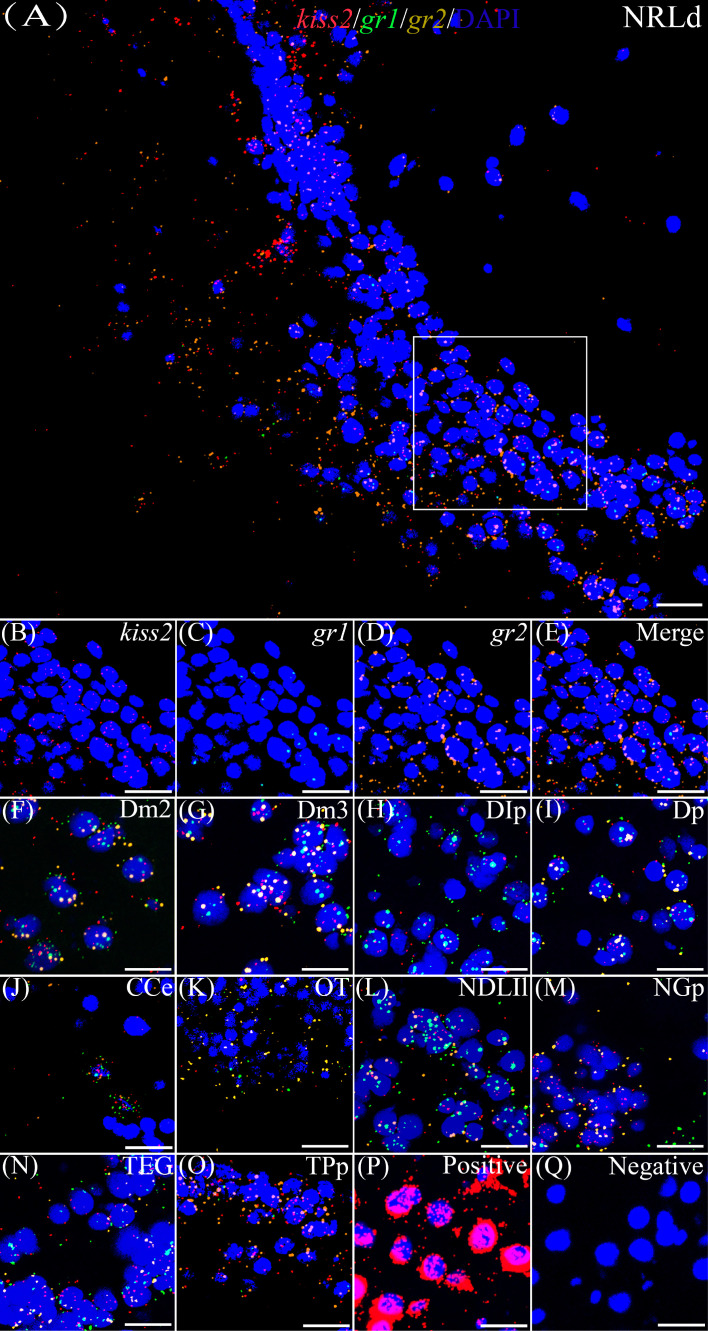
Co-expression of *gr1*, *gr2*, and *kiss2*. The view of NRLd **(A)** shows *kiss2* (red), *gr1* (green), and *gr2* (yellow) with DAPI cell nuclear staining (blue). **(B–E)** The view of boxed region in panel **(A)**, showing *kiss2* (red), *gr1* (green), and *gr2* (yellow) with DAPI cell nuclear staining (blue) in panels **(B–D)**, respectively; and the “merge” in panel **(E)** shows *kiss2* (red), *gr1* (green), and *gr2* (yellow) with DAPI cell nuclear staining (blue). Representative images display *gr1* (green) and *gr2* (yellow) co-expression of *kiss2* (red) with DAPI cell nuclear staining (blue) in Dm2 **(F)**, Dm3 **(G)**, DIp **(H)**, Dp **(I)**, CCe **(J)**, OT **(K)**, NDLIl **(L)**, NGp **(M)**, TEG **(N)**, and TPp **(O)**. Positive and negative controls are shown in panels **(P**, **Q)**, respectively. NRLd, dorsal part of the nucleus of the lateral recess; Dm2, subdivision 2 of the medial dorsal telencephalic area; Dm3, subdivision 3 of the medial dorsal telencephalic area; DIp, lateral posterior part of the dorsal telencephalic area; Dp, posterior portion of the dorsal telencephalon; CCe, corpus of the cerebellum; OT, optic tectum; NDLIl, lateral part of the diffuse nucleus of the inferior lobe; NGp, posterior part of glomerular nucleus; TEG, tegmentum; TPp, periventricular nucleus of the posterior tuberculum. Bars = 20 μm.

According to the distribution of *kiss1* and *kiss2* genes in the brain of yellowtail clownfish (unpublished), RNAscope *in situ* hybridization for *gr1* and *gr2* and *kiss1/kiss2* genes were performed in areas of the telencephalon (Te), mesencephalon (Me), cerebellum (Ce), and hypothalamus (Hy). The co-expression of *gr1*, *gr2*, and *kiss1* was found in the in the dorsal habenular nucleus (NHd), subdivision 2 of the medial dorsal telencephalic area (Dm2), subdivision 3 of the medial dorsal telencephalic area (Dm3), lateral posterior part of the dorsal telencephalic area (DIP), posterior portion of the dorsal telencephalon (DP), corpus of the cerebellum (CCe), and lateral part of the diffuse nucleus of the inferior lobe (NDLIl) (**Figures 2A–E, F–K**). The *gr2* signal was more abundantly distributed than *gr1* in NHd, Dm2, and CCe (**Figures 2A, F, J**) and weakly expressed in the DIP and DP (**Figures 2H, I**). The *gr1*, *gr2*, and *kiss2* were simultaneously detected in the dorsal part of the nucleus of the lateral recess (NRLd), Dm2, Dm3, DIP, DP, CCe, optic tectum (OT), NDLIl, posterior part of glomerular nucleus (NGP), tegmentum (TEG), and periventricular nucleus of the posterior tuberculum (TPp) (**Figures 3A–E, F–O**). Compared with *gr1*, the stronger *gr2* signals were detected in the NRLd, Dm2, Dm3, OT, NGP, and TPp (**Figures 3A, F, G, K, M, O**), but the weaker signaling molecules were examined in the DIP and NDLIl (**Figures 3H, L**).

### *In silico* analysis of 5′-flanking region for the yellowtail clownfish *kiss2* gene

**Figure 4:**
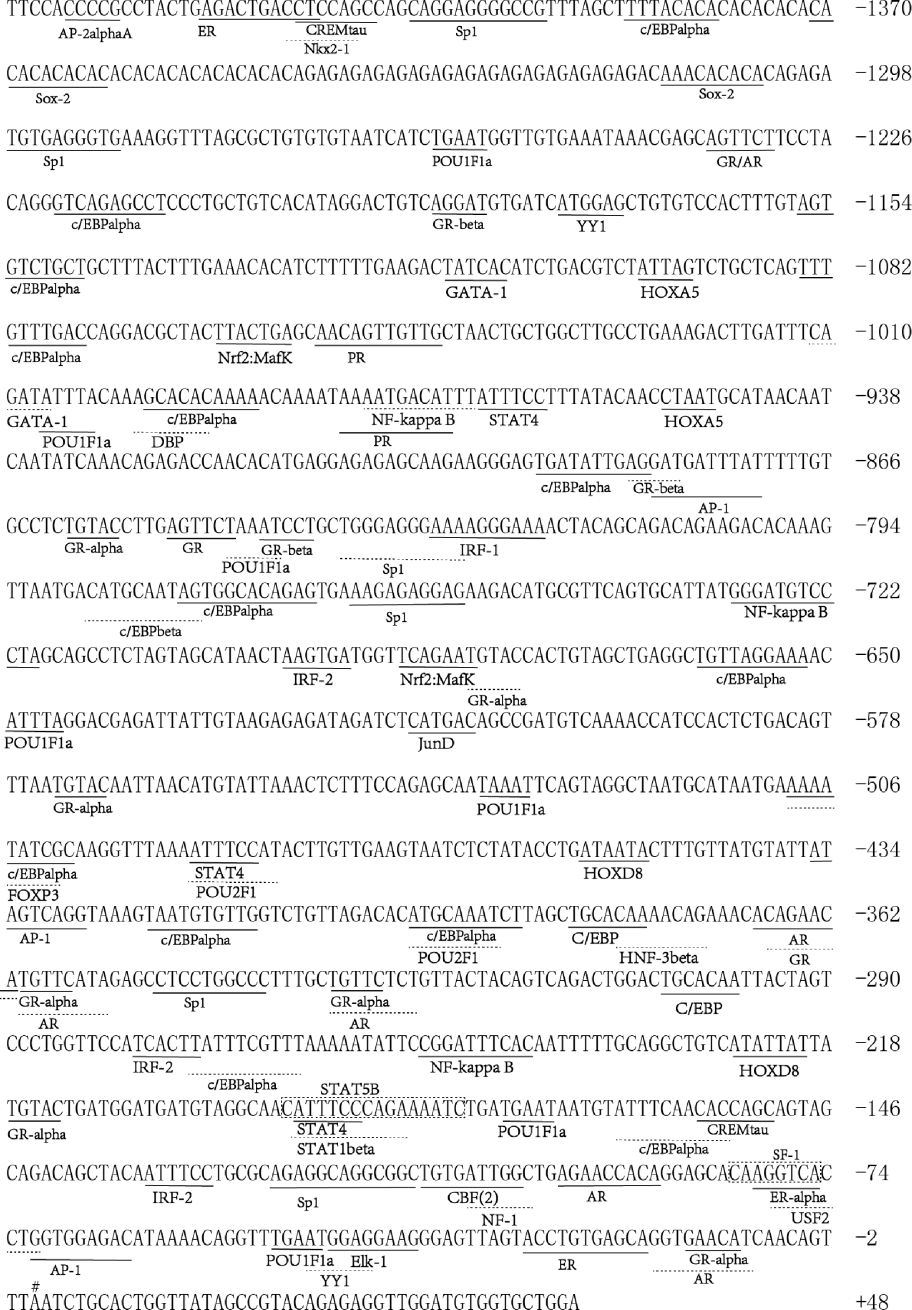
Sequence analysis of 5′-flanking region for *kiss2*. The numbering of the sequence is relative to the transcription start site marked on # and designated as +1. Putative binding sites for transcription factors are underlined and labeled. Transcription factor binding sites were predicted using the online PROMO and gene-regulation tool.

To further investigate the transcriptional regulatory mechanism of *kiss2* by cortisol in yellowtail clownfish, we isolated the 5′-flanking region of *kiss2* gene. The putative *kiss2* promoter sequence includes a 1,442-bp upstream of the transcription start site and a 48-bp first exon fragment (**Figure 4**). *In silico* analysis revealed that *kiss2* promoter sequence possessed 13 potential glucocorticoid response elements (GREs). In addition, several motifs for other steroid receptors were identified on the *kiss2* promoter, such as five androgen response elements, three estrogen response elements, and two progesterone response elements (**Figure 4**).

### Effects of cortisol on *kiss2* promoter activity

**Figure 5:**
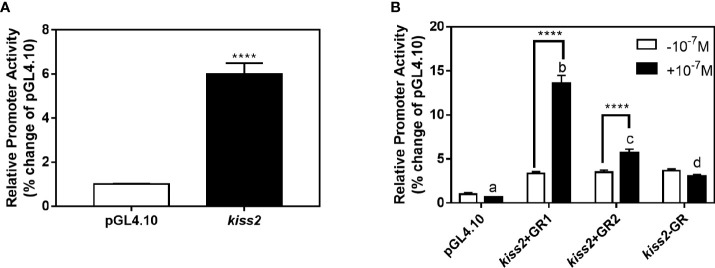
Effects of cortisol on *kiss2* promoter activity. **(A)** Basic activity of *kiss2* gene promoter in HEK-293T cell lines. The cells were transfected with 0.5 μg pkiss2-1442 and 0.025 μg pRL-CMV, or 0.5 μg pGL4.10 and 0.025 μg pRL-CMV as the control. Luciferase activity was measured after 24 h. Relative promoter activities are expressed as percentage of pGL4.10. **(B)** The activities of *kiss2* promoter in the presence of cortisol in HEK293T cell lines. Cells were co-transfected with 0.5 μg pkiss2-1442, and 0.025 μg pRL-CMV with or without 0.05 μg yellowtail clownfish glucocorticoid receptor (GR1 or GR2) expression plasmid. The transfected cells were treated with or without 10^−7^ M cortisol. The luciferase activity was measured 24 h later. Relative promoter activities are expressed as percentage of pGL4.10 in the absence of cortisol. Data are represented as mean ± SEM (n = 4). **** (p < 0.0001) indicates that significant difference compared with the corresponding control. The different letters mean significant differences between groups with cortisol treatment (p < 0.05).

**Figure 6:**
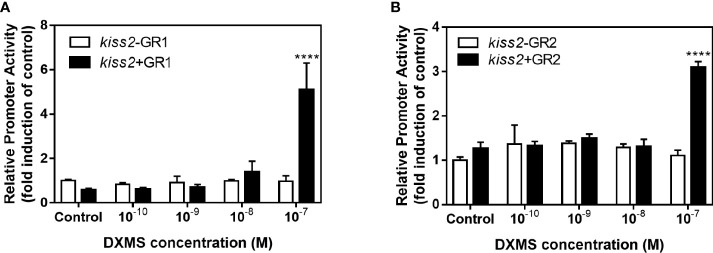
Effects of cortisol with different concentrations on the activities of *kiss2* gene promoter. HEK-293T cells were transfected with 0.5 μg pkiss2-1442, 0.05 μg GR1 **(A)** or GR2 **(B)** and 0.025 μg pRL-CMV; 0.5 μg pGL4.10 co-transfected with 0.025 μg pRL-CMV as the control. Cells were treated with or without cortisol. Luciferase activity was detected after 24 h. Data are represented as mean ± SEM (n = 4). **** (p < 0.0001) indicates significant differences between groups with cortisol treatment.

GR-negative HEK293T cells were transfected with the recombinant vector for *kiss2* promoter (pkiss2-1442), in combination with expression plasmids for yellowtail clownfish glucocorticoid receptor (GR1 or GR2), to analyze the transcriptional regulation of *kiss2* by cortisol. Basal promoter activity was examined for the *kiss2* promoter, indicating that functional promoter activity existed in the 5′-flanking region (**Figure 5A**). DXMS significantly increased *kiss2* promoter activity in the presence of GR, being more efficient in GR1 than GR2 (**Figure 5B**). The *kiss2* promoter activities were detected after treatment with different DXMS concentrations and significantly upregulated at 10^−7^ M DXMS in the presence of GR1 or GR2 (**Figures 6A, B**). Therefore, this concentration of DXMS was chosen for the subsequent investigation.

### Identification of glucocorticoid-responsive region and functional GRE site on the yellowtail clownfish *kiss2* promoter

**Figure 7:**
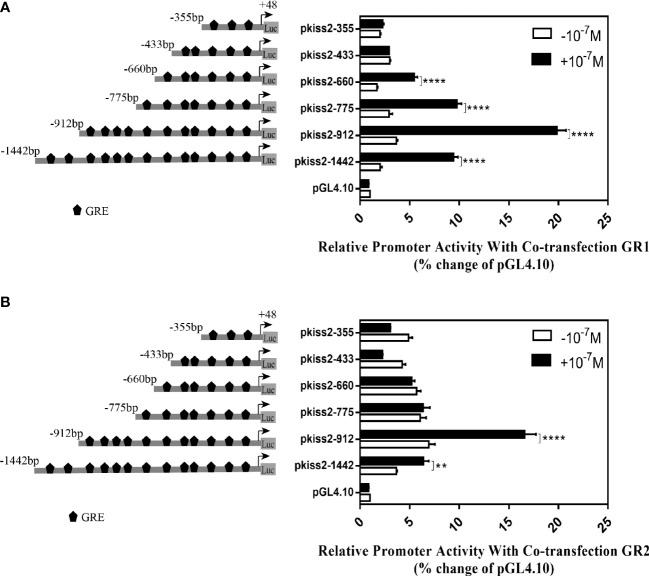
Deletion analysis of the *kiss2* promoter. Schematic of the putative GRE sites and deletion constructs of *kiss2* promoter is shown on the left. HEK-293T cells were transfected with deletion constructs and pRL-CMV with GR1 **(A)** or GR2 **(B)** expression plasmid. Cells were incubated with or without 10^−7^ M cortisol treatment. Relative promoter activities are expressed as percentage of pGL4.10 in the absence of cortisol. Data are represented as mean ± SEM (n = 4). **(p < 0.01) and **** (p < 0.0001) indicate the significant differences compared with the corresponding control.

**Figure 8:**
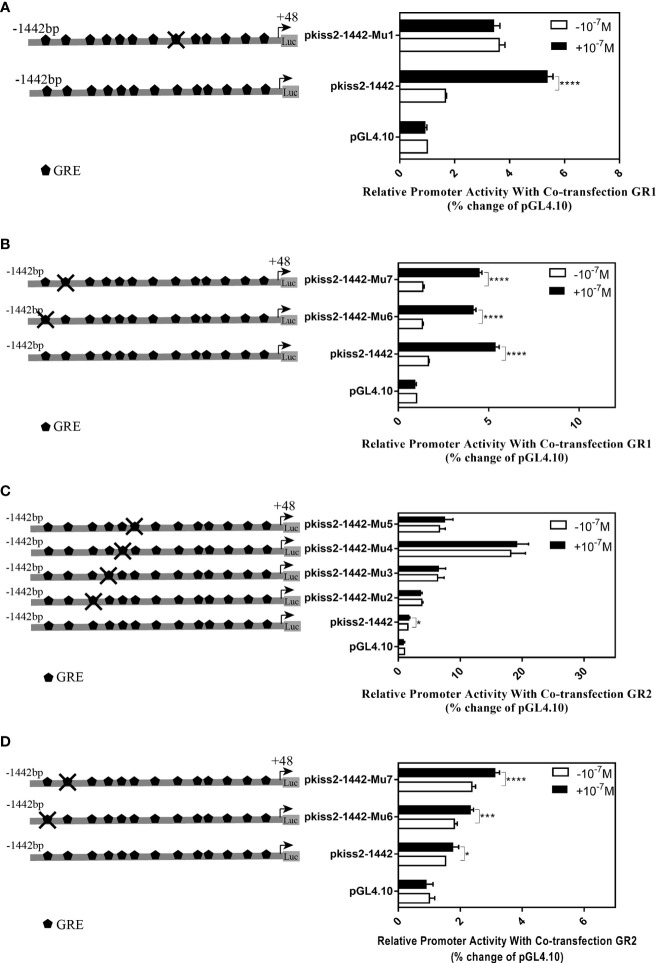
Mutations of the putative glucocorticoid receptor binding sites in *kiss2* promoter. Schematic of mutated GRE sites is shown on the left. HEK-293T cells were transfected with GRE-mutated promoter constructs and pRL-CMV with GR1 **(A, B)** or GR2 **(C, D)** expression plasmid. Cells were incubated with or without 10^−7^ M cortisol treatment. Relative promoter activities are expressed as percentage of pGL4.10 in the absence of cortisol. Data are represented as mean ± SEM (n = 4). * (p < 0.05), *** (p < 0.001), and **** (p < 0.0001) indicate significant differences compared with the corresponding control.

Using the full length of the *kiss2* promoter vector as a template, a series of deletion constructs were established as shown in the left panel of **Figures 7**, **8**. In the presence of GR1, the promoter activity of *kiss2* was significantly higher after 10^−7^ M DXMS treatment (**Figure 7A**). Deletions of *kiss2* promoter to position −433 (pkiss2-433) abolished cortisol-induced promoter activity, indicating that the region from −660 to −433 is relevant with cortisol-induced promoter activity by GR1 (**Figure 7A**). Site-directed mutagenesis was conducted to determine whether the GRE at −573 (^−573^ TGTAC^−569^) was the key regulatory site on *kiss2* promoter. Mutation of a GRE at −573 eradicated cortisol-induced promoter activity (**Figure 8A**). However, we found that mutation in other GRE sites at −1,236 (^−1,236^AGTTCT^−1,231^) or −1,188 (^−1,188^AGGAT^−1,184^) did not change cortisol-induced promoter activity (**Figure 8B**). Thus, the GRE at −573 is critical for cortisol/GR1-induced *kiss2* promoter activity.

In the presence of GR2, 10^−7^ M DXMS treatment significantly upregulated the wild-type *kiss2* promoter activity (**Figure 7B**).Truncation of the *kiss2* promoter to −775 bp abolished by cortisol-induced promoter activity (**Figure 7B**). Mutations of two GRE at −1,236 or −1,188 of the *kiss2* promoter still responded to the cortisol treatment (**Figure 8D**). However, cortisol-induced promoter activities of *kiss2* were removed in mutations of the following four GRE at −883 (^−883^AGGAT^−879^), −860 (^−860^TGTAC^−856^), −851 (^−851^AGTTCT^−846^), and −843 (^−843^ATCCT^−839^), indicating that these GRE binding sites contribute to cortisol-/GR2-induced *kiss2* promoter activity (**Figure 8C**).

## Discussion

Previous studies in vertebrates have demonstrated that kisspeptin plays a vital role in mediating the stress-induced reproductive regulation (7). Cortisol, the main steroid hormone for stress response, is involved in gonadal development and sexual differentiation by regulating *kiss* genes (26). This study aims to observe the molecular mechanism of glucocorticoid regulation of the *kiss* genes in teleosts. In rodents, the hypothalamic *kiss1* mRNA level and kisspeptin neuron activity are reduced after the administration of cortisol (16, 17). There is only one *kiss* gene in rodents, but there are two *kiss* genes (*kiss1* and *kiss2*) in several teleost fish participating in the reproductive regulation (2). Cortisol treatment showed that the expression of *kiss* (*kiss1* and *kiss2*) and *gr* (*gr1* and *gr2*) genes were increased significantly in the brain of yellowtail clownfish. In addition, elevation of two *kiss* genes expression by cortisol has been reported in zebrafish (29). Moreover, our previous study revealed that *kiss2*, E_2_, and testosterone (T) levels are higher in the subordinate than in the dominant yellowtail clownfish (25). A study in protandrous false clown anemonefish (*Amphiprion ocellaris*) showed that the social rank reflects the blood cortisol value (30).

GR signals are detected in the Kiss neurons of periventricular nucleus continuum (AVPV/PeN) and arcuate nucleus (ARC) by double-labeling immunohistochemistry in female rats (19). In the present study, we have demonstrated that Kiss neurons co-expressed the glucocorticoid receptors in the Te, Me, Ce, and Hy, suggesting that cortisol could directly affect kisspeptin neurons *via* GR in yellowtail clownfish. Habenular *kiss1* and serotonin-related genes are downregulated after exposure to alarm substance (AS), and Kiss1 antagonist injection can reduce AS-evoked fish fear response, indicating that habenular kisspeptin modulates fear in zebrafish (31). The yellowtail clownfish non-breeders are always attacked by both female and male, and hypothalamic *gr2* levels of non-breeders are significantly higher than that of the others in one social group (21, 26). From our results showing *gr* genes co-expression with *kiss1* in NHd, we raise the possibility that habenular *kiss1* is involved in the regulation of fear response in the yellowtail clownfish. Moreover, *kiss2* levels in the NRLd are decreased under testosterone (T) treatment in sea bass (32). In yellowtail clownfish, *gr1* and *gr2* were found to be co-expressed with *kiss2* in the NRLd, indicating that this region may participate in stress-induced reproductive functions *via kiss2*.

Multiple GR binding sites were found in *kiss2* promoter in the yellowtail clownfish by silicon analysis, implying that *kiss2* could be regulated by cortisol *via* GR through binding with GRE. In the present study, cortisol injection also enhanced the *gr1* and *gr2* mRNA levels in the brain. Other binding sites such as AP1, Sp1, and C/EBP were also predicted in the *kiss2* promoter region. AP1 could interact with GR for GR-regulated transcription and recruitment to co-occupied AP1 binding site by DNaseI accessibility and chromatin immunoprecipitation with high-throughput sequencing (33). In addition, a series of binding sites for sex steroid receptors existed in the *kiss2* promoter of yellowtail clownfish, which is similar to the results of goldfish and zebrafish (20, 34), indicating that the potential ability of *kiss2* is involved in the regulation of reproduction. Our previous study found that hypothalamic *kiss2* had higher expression in non-breeders than females and males, which may contribute to the regulation of gonad development under social stress in the yellowtail clownfish (25).

The cortisol treatment could enhance yellowtail clownfish *kiss2* promoter activities in HEK293T cells in the presence of GR, whereby GR1 was more effective than GR2. In yellowtail clownfish, GR1 contains conserved nine amino acids, which are present in the most known teleostean GR1 proteins but absent in other vertebrates (26, 35, 36). A previous study revealed that the additional nine amino acids made GR1a to better bind with single GRE than GR1b in rainbow trout (*Oncorhynchus mykiss*) (37). Thus, we speculate that GR1 has a better binding affinity for GRE than GR2 in the yellowtail clownfish. Altogether, GRs play a vital role in the mediation of cortisol effect on *kiss2* promoter.

GR can activate or repress gene expression by binding with GRE directly or interacting with other transcription factors (38). Using a series of deletion constructs, we have demonstrated that cortisol-induced promoter activities of *kiss2* gene were located between position −660 and −433 with GR1, and −912 and −775 with GR2, respectively. Point mutations in the *kiss2* promoter were generated by site-directed mutagenesis. Our results showed that in the presence of GR1, cortisol-stimulated promoter activity was only mediated by one GRE site located at the position of −573, whereas in the presence of GR2, promoter activity could be modulated by all four GRE sites located at positions −883, −860, −851, and −843. Therefore, the *kiss2* gene is regulated by cortisol through the GRE-dependent mechanism in yellowtail clownfish. It has also been reported that there is a synergistic action between the enhancer binding protein (C/EBP) and GR in the regulation of thymidine kinase promoter activity (39).

In conclusion, the present study demonstrated for the first time the molecular mechanism of glucocorticoid regulation of the *kiss* genes in teleosts. It was found that cortisol treatment could upregulate the expression levels of *kiss* and *gr* genes in the yellowtail clownfish. The Kiss neurons coexpressed the glucocorticoid receptors in Te, Me, Ce, and Hy. Cortisol could enhance *kiss2* promoter activity in the presence of GRs and was more effective with GR1 than GR2. Moreover, cortisol was shown to regulate *kiss2* promoter activity by one GRE site through GR1 and four GRE sites *via* GR2. Our findings demonstrate that cortisol could directly regulate the expression of *kiss2* gene *via* the GRE-dependent GR pathway in the yellowtail clownfish.

## Data availability statement

The original contributions presented in the study are included in the article/**Supplementary Material**. Further inquiries can be directed to the corresponding author.

## Ethics statement

The animal study was reviewed and approved by Committee of Hainan University (HNUAUCC-2021-00014).

## Author contributions

S-YB planned and wrote the manuscript, participated in experiments and composed the figures. Y-YZ planned, edited, and drafted the manuscript. XZ, T-XL, D-CZ, and Z-XH participated in experiments. QW planned, edited, supervised, and reviewed the manuscript. All authors read the final article and approved its submission.

## Funding

This work was supported by National Natural Science Foundation of China (NSFC Grant No. 31760759).

## Conflict of interest

The authors declare that the research was conducted in the absence of any commercial or financial relationships that could be construed as a potential conflict of interest.

## Publisher’s note

All claims expressed in this article are solely those of the authors and do not necessarily represent those of their affiliated organizations, or those of the publisher, the editors and the reviewers. Any product that may be evaluated in this article, or claim that may be made by its manufacturer, is not guaranteed or endorsed by the publisher.
