# Supplementary material for: Regulation of the kiss2 promoter in yellowtail clownfish (Amphiprion clarkii) by cortisol via GRE-dependent GR pathway
Source: Front Endocrinol (Lausanne). 2022 Aug 3;13:902737. doi: 10.3389/fendo.2022.902737 (PMC9382246; doi:10.3389/fendo.2022.902737)
Supplement: Supplementary file 1 [file Table_1.docx]

**Table S1**

Primers used in the present study.

| Primers | Primer efficiency | Sequences（from 5’ to 3’） |
| --- | --- | --- |
| **Primers for qPCR** | | |
| *kiss1*-qpcr-F | 107.4% | CCACCGCTCATTGTTGTTC |
| *kiss1*-qpcr-R |  | CATCCTGGCCTGGGAAATA |
| *kiss2*-qpcr-F | 103.1% | TTCGCTGATGGTTGGGC |
| *kiss2*-qpcr-R |  | CTTACTCCTGCGGTCGTTGC |
| *gr1*-qpcr-F | 108.2% | TCAAAGATGGGAGCGTCAAG |
| *gr1*-qpcr-R |  | ATGAGGGAGAGGCAGGATAA |
| *gr2*-qpcr-F | 107.7% | TTGACCGAACTGGGACACTG |
| *gr2*-qpcr-R |  | CTAAGGGGGTGGTTGGGTGAG |
| β-actin-F | 104.9% | ACAGTGCCCATCTACGAGG |
| β-actin-R |  | GCTGTGGTGGTGAAGGAATA |
| **Primers for genome walking** | | |
| GW-AP1 |  | GTAATACGACTCACTATAGGGC |
| GW-AP2 |  | ACTATAGGGCACGCGTGGT |
| *kiss2*-SP1 |  | ATCTGCTGCCACACTCCCTCCATCCT |
| *kiss2*-SP2 |  | ATCGTCCAGCACCACATCCAACCTCT |
| **Construction of recombinant vector** | | |
| *kiss2*-1442-F |  | CTAGGTACCTTCCACCCCGCCTACTGAGACT |
| *kiss2*-1442-R |  | CCCCTCGAGTCCAGCACCACATCCAACCTCT |
| *kiss2*-883-F |  | CTAGGTACCGAGGAGAGAGCAAGAAGGGAGT |
| *kiss2*-681-F |  | CTAGGTACCGGCACAGAGTGAAAGAGAGGAGA |
| *kiss2*-573-F |  | CGGGGTACCGTTAGGAAAACATTTAGGACGAG |
| *kiss2*-367-F |  | CGGGGTACCAGTCAGGTAAAGTAATGTGTTGG |
| *kiss2*-333-F |  | CTAGGTACCATAGAGCCTCCTGGCCCTTTG |
| **Primers for site-directed mutagenesis** | | |
| *kiss2*-GREmu-1236-F |  | ATAAACGAGCGCCCGCTCCTACAGGGTCAGA |
| *kiss2*-GREmu-1236-R |  | TCTGACCCTGTAGGAGCGGGCGCTCGTTTAT |
| *kiss2*-GREmu-1188-F |  | AGGACTGTCGCCGGGTGATCATGGAGCTGT |
| *kiss2*-GREmu-1188-R |  | ACAGCTCCATGATCACCCGGCGACAGTCCT |
| *kiss2*-GREmu-883-F |  | GAGAGCAAGAAGGGAGTGATATTGGCCGGGATTT |
| *kiss2*-GREmu-883-R |  | AAATCCCGGCCAATATCACTCCCTTCTTGCTCTC |
| *kiss2*-GREmu-860-F |  | GTGCCTCGCCGGCTTGAGTTCTAAATCCTGCT |
| *kiss2*-GREmu-860-R |  | AGCAGGATTTAGAACTCAAGCCGGCGAGGCAC |
| *kiss2*-GREmu-851-F |  | ACCTTGGCCGGTAAATCCTGCTGGGAGGGAAAA |
| *kiss2*-GREmu-851-R |  | TTTTCCCTCCCAGCAGGATTTACCGGCCAAGGT |
| *kiss2*-GREmu-843-F |  | GTGCCTCTGTACCTTGAGTTCTAAGCCGGGCT |
| *kiss2*-GREmu-843-R |  | AGCCCGGCTTAGAACTCAAGGTACAGAGGCAC |
| *kiss2*-GREmu-573-F |  | CCATCCACTCTGACAGTTTAAGCCGGAATTAACAT |
| *kiss2*-GREmu-573-R |  | ATGTTAATTCCGGCTTAAACTGTCAGAGTGGATGG |
